# Supplementary material for: A Critical View of the Application of the APEX Software (Aqueous Photochemistry of Environmentally-Occurring Xenobiotics) to Predict Photoreaction Kinetics in Surface Freshwaters
Source: Molecules. 2019 Dec 18;25(1):9. doi: 10.3390/molecules25010009 (PMC7017383; doi:10.3390/molecules25010009)
Supplement: Supplementary file 1 [file molecules-25-00009-s001.zip › 3.2.4_gcc-4.4.0.zip!/3.2.4_gcc-4.4.0/3.2.4_gcc-4.4.0/doc/octave/html/interpreter/Printing-Plots.html]

Printing Plots - Untitled


Next: Interacting with plots,
Previous: Multiple Plot Windows,
Up: Plotting Basics


---

#### 15.1.6 Printing Plots

The `print` command allows you to save plots in a variety of
formats. For example,

```
     print -deps foo.eps
```

writes the current figure to an encapsulated PostScript file called
foo.eps.

— Function File: **print** ()  
— Function File: **print** (options)  
— Function File: **print** (filename, options)  
— Function File: **print** (h, filename, options)  
> Print a graph, or save it to a file
>
> filename defines the file name of the output file. If no
> filename is specified, the output is sent to the printer.
>
> h specifies the figure handle. If no handle is specified
> the handle for the current figure is used.
>
> options:
>
> `-P`printer: Set the printer name to which the graph is sent if no filename is specified. `-G`ghostscript\_command: Specify the command for calling Ghostscript. For Unix and Windows, the defaults are 'gs' and 'gswin32c', respectively. `-color` `-mono`: Monochrome or color lines. `-solid` `-dashed`: Solid or dashed lines. `-portrait` `-landscape`: Specify the orientation of the plot for printed output. `-d`device: Output device, where device is one of: `ps` `ps2` `psc` `psc2`: Postscript (level 1 and 2, mono and color) `eps` `eps2` `epsc` `epsc2`: Encapsulated postscript (level 1 and 2, mono and color) `tex` `epslatex` `epslatexstandalone` `pstex` `pslatex`: Generate a LaTeX (or TeX) file for labels, and eps/ps for graphics. The file produced by `epslatexstandalone` can be processed directly by LaTeX. The other formats are intended to be included in a LaTeX (or TeX) document. The `tex` device is the same as the `epslatex` device. `ill` `aifm`: Adobe Illustrator `cdr` `corel`: CorelDraw `dxf`: AutoCAD `emf` `meta`: Microsoft Enhanced Metafile `fig`: XFig. If this format is selected the additional options `-textspecial` or `-textnormal` can be used to control whether the special flag should be set for the text in the figure (default is `-textnormal`). `hpgl`: HP plotter language `mf`: Metafont `png`: Portable network graphics `jpg` `jpeg`: JPEG image `gif`: GIF image `pbm`: PBMplus `svg`: Scalable vector graphics `pdf`: Portable document format If the device is omitted, it is inferred from the file extension, or if there is no filename it is sent to the printer as postscript. `-d`gs\_device: Additional devices are supported by Ghostscript. Some examples are; `ljet2p`: HP LaserJet IIP `ljet3`: HP LaserJet III `deskjet`: HP DeskJet and DeskJet Plus `cdj550`: HP DeskJet 550C `paintjet`: HP PointJet `pcx24b`: 24-bit color PCX file format `ppm`: Portable Pixel Map file format For a complete list, type `system ("gs -h")' to see what formats and devices are available. When the ghostscript is sent to a printer the size is determined by the figure's "papersize" property. When the ghostscript output is sent to a file the size is determined by the figure's "paperposition" property. `-r`NUM: Resolution of bitmaps in pixels per inch. For both metafiles and SVG the default is the screen resolution, for other it is 150 dpi. To specify screen resolution, use "-r0". `-tight`: Forces a tight bounding box for eps-files. Since the ghostscript devices are conversion of an eps-file, this option works the those devices as well. `-S`xsize`,`ysize: Plot size in pixels for EMF, GIF, JPEG, PBM, PNG and SVG. If using the command form of the print function, you must quote the xsize,ysize option. For example, by writing `"-S640,480"`. The size defaults to that specified by the figure's paperposition property. `-F`fontname `-F`fontname`:`size `-F:`size: fontname set the postscript font (for use with postscript, aifm, corel and fig). By default, 'Helvetica' is set for PS/Aifm, and 'SwitzerlandLight' for Corel. It can also be 'Times-Roman'. size is given in points. fontname is ignored for the fig device.
>
> The filename and options can be given in any order.

— Function File: **orient** (orientation)  
> Set the default print orientation. Valid values for
> orientation include `"landscape"`, `"portrait"`,
> and `"tall"`.
>
> The `"tall"` option sets the orientation to portait and fills
> the page with the plot, while leaving a 0.25in border.
>
> If called with no arguments, return the default print orientation.
